# Food frequency questionnaire for adults in the Brazilian Northeast region: emphasis on the level of food processing

**DOI:** 10.11606/s1518-8787.2021055002473

**Published:** 2021-08-06

**Authors:** Virginia Williane de Lima Motta, Severina Carla Vieira Cunha Lima, Dirce Maria Lobo Marchioni, Clélia de Oliveira Lyra

**Affiliations:** I Universidade Federal do Rio Grande do Norte Centro de Ciências da Saúde Programa de Pós-Graduação em Nutrição NatalRN Brasil Universidade Federal do Rio Grande do Norte. Centro de Ciências da Saúde. Programa de Pós-Graduação em Nutrição. Natal, RN, Brasil; II Universidade Federal do Rio Grande do Norte Centro de Ciências da Saúde Departamento de Nutrição NatalRN Brasil Universidade Federal do Rio Grande do Norte. Centro de Ciências da Saúde. Departamento de Nutrição. Natal, RN, Brasil; III Universidade de São Paulo Faculdade de Saúde Pública Departamento de Nutrição São PauloSP Brasil Universidade de São Paulo. Faculdade de Saúde Pública. Departamento de Nutrição. São Paulo, SP, Brasil

**Keywords:** Adult, Food Consumption, Industrialized Foods, Surveys and Questionnaires, Noncommunicable Diseases, prevention & control

## Abstract

**OBJECTIVE:**

To develop a quantitative Food Frequency Questionnaire (FFQ) for adults in the Northeast region of Brazil, in order to identify the frequency of consumption of foods considered to be of protection and risk for chronic non-communicable diseases (NCDs), grouping food items by processing level.

**METHODS:**

To develop the FFQ, data from 7,516 adults from Northeastern Brazil were used, extracted from the 2008–2009 Household Budget Survey. The food lists were elaborated according to the methodology of the relative contribution of each item, identifying foods with the highest relative contribution for macronutrients, fiber, saturated fat, trans fat, sodium and potassium. All foods whose contribution sum was up to 90% composed such lists. The final structure of the FFQ organized the food items in order to respect the mental image of the meals.

**RESULT:**

The FFQ resulted in 83 food items, distributed in minimally processed, processed and ultra-processed. We chose the previous year as the time to estimate food consumption, and frequency options ranged from “never” to “10 times”. The instrument includes guidelines for filling and collects data on serving sizes (small, medium, large and extra-large), as well as additional information on culinary preparations. There was a high percentage of people who were overweight (44.1%).

**CONCLUSION:**

The study culminated in an FFQ to identify the frequency of consumption of foods considered protective and risk for NCDs. The instrument can support epidemiological studies that evaluate outcomes related to the diet of adults considering the level of food processing, in accordance with the *Guia alimentar para a população brasileira* .

## INTRODUCTION

The Food Frequency Questionnaire (FFQ) is a food survey method used in epidemiological investigations to collect information on food and dietary consumption. Its goal is to investigate the relation between diet and disease^1^ .

The FFQ, whose precursor is the checklist developed by Burke^2^ , has among its advantages: ability to evaluate the usual diet without changing the pattern of food consumption, low cost and shorter filling time compared to the food record^1^ . The limitation of the instrument, on the other hand, lies in the fact that it documents the food intake of individuals within a certain period of time, which can lead to reports distorted by memory bias, and it also shows a low accuracy when quantifying the diet^3^ .

To minimize the limitations of an FFQ, it is necessary to follow the appropriate methodology rigorously in order to obtain accurate and economically viable instruments. According to the objective of the study, the researcher should systematize the items that compose the questionnaire, such as food list, categories of frequency of consumption and type of questionnaire: qualitative, semi-quantitative or quantitative^4^ . The qualitative type does not include serving size, while the semi-quantitative does. The quantitative includes reference sizes: small, medium and large^1^ .

In Brazil, the traditional diet is marked by the intake of foods such as rice, beans and fruits. However, we have been observing changes in this diet in all age groups^5^ . In natura or minimally processed foods have been replaced by ultra-processed foods^6^ .

These changes in the traditional Brazilian diet are accompanied by an increase in the prevalence of diabetes, obesity and other chronic non-communicable diseases (NCDs). An inadequate diet, with high consumption of ultra-processed foods (products rich in salt, saturated fats, trans fats and sugars)^7^ , is one of the modifiable risk factors related to NCDs worldwide^8^ .

Although the Northeast region, studied here, stands out for its characteristic cuisine, with regional foods considered good sources of various nutrients^9^ , the northeastern population has shown high prevalence of NCDs^10^ . Therefore, it is important to develop a specific questionnaire for the region, capable of monitoring the relation between food consumption and health-disease process with a focus on the level of food processing.

The *Guia alimentar para a população brasileira* (Food guide for the Brazilian population)^11^ directs food consumption recommendations according to the new classification; in this context, epidemiological studies could use the FFQ to evaluate the adherence of the recommendations by the population. However, there are still limited studies that seek to establish the relation between consumption of ultra-processed foods and health, since there are no specific instruments to evaluate the consumption of these products, and traditional instruments have not been developed for this purpose^12^ .

Thus, this study aimed to develop a quantitative FFQ for adults in the Northeast region of Brazil, in order to identify the frequency of consumption of foods considered to be of protection and risk for NCDs. The instrument grouped food items by processing level.

## METHODS

This study uses personal food consumption data from the National Food Survey (INA) 2008–2009, a module of the Household Budget Survey (HBS) in which 34,003 individuals aged 10 years or over participated. For two consecutive days, participants completed food records (FR), in which they noted time and place of food consumption, quantities in homemade measures and form of preparation. The other details about sampling and data collection of HBS are published in official research document^5^ .

To compose the sample of this study, 7,516 people from the Northeast region were considered, between 20 and 59 years of age, who completed the food records in the HBS. Pregnant and lactating women (n = 419) were excluded. Since the study used a secondary database in the public domain, submission to the Research Ethics Committee was unnecessary.

### Database Construction and Analysis

Research data were obtained in the *Sistema IBGE de Recuperação Automática* (SIDRA – IBGE Automatic Recovery System), by downloading microdata with coded information of all the residents of the households that participated in the 2008–2009 HBS. To import and read the data, the statistical package data Zoom, Stata version 12 for Windows was used^a^ .

To obtain the list of foods, two databases were accessed: one referring to the characteristics of individuals (RECORD: PEOPLE - HBS 1) and another with information on individual food consumption (RECORD: FOOD CONSUMPTION - HBS 7). The consumed quantities of the food were transformed into grams or milliliters, based on the table of measures referred to for food consumed in Brazil of the 2008–2009 HBS ^13^ .

The information obtained by the sum of the food records of the sample represented 153,617 food data, that is, all the foods consumed by the sample in the two days of registration. We chose to add the foods recorded in the two days in order to include as many foods as possible most often consumed by the population in question. Then, 1,149 foods were identified, in their various forms of preparation (for example, “cooked chicken” and “roasted chicken”).

The consumption of energy, macronutrients, fiber, saturated fat, trans fat, sodium and potassium was estimated from the *Tabela de Composição Nutricional dos Alimentos Consumidos no Brasil* by HBS^14^ . Nutrients were chosen considering food characteristics related to protection or risk for NCDs^15 , 16^ . In addition to the nutrients mentioned above, a component considered critical for NCDs is free sugars^15 , 16^ . In the HBS data, however, there is no information about this component. Thus, its evaluation is impossible.

In the next step, a code was assigned per food, regardless of the forms of preparation, with the exception of fried meats, which remained separate. Despite the limitations of the Nutritional Composition Table, especially regarding the lack of information on trans fat according to the preparation, we chose to keep the fried preparations separate, given the difference in fat content.

Subsequently, the values of nutrients of interest of foods with equal codes were added. For example: the calories of cooked chicken were added to the calories of roasted chicken, and both coded as chicken. At the end of this process, the 1,149 foods were reduced to 778 foods.

**Figure 1:**
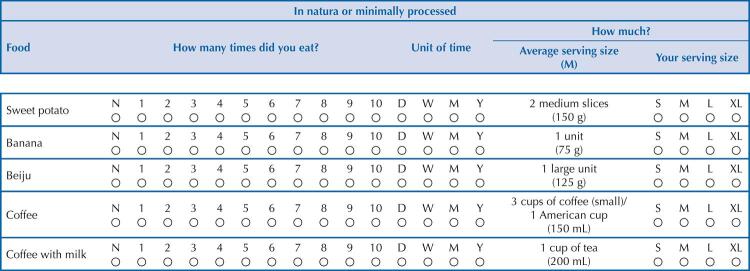
Flowchart of the organization of the database for the construction of a Food Frequency Questionnaire.

Foods that did not have a specific description (e.g., unspecified soda), or had a description similar to that of another food (e.g., ground meat and meatball), were included in a single item, by equivalence. The need to group poorly consumed foods (cited less than 20 times^17^ ) in a single item was also necessary, considering the similarity between them. Thus, three new groups were created: “Other fruits”, “Other cheeses” and ”Other alcoholic beverages”. Regarding beef and chicken, these were aggregated according to the characteristic “with bone” and “without bone”, given the difference in the amount of fat. After all adjustments, 421 foods were considered for the FFQ list ( Figure 1 ).

N: never; D: Day; W: Week; M: month; Y: year; S: Small; M: medium; L: large; XL: extra large.

### Food List

To construct the lists of foods based on the nutrients of interest, we opted for the methodology of Block et al., which considers the relative contribution of the item^17^ , identifying the items with the greatest relative contribution for macronutrients, fiber, saturated fat, trans fat, sodium and potassium. The lists were composed of all foods whose contribution sum was up to 90%, as established in other studies^18 , 19^ .

In this study, no more than 100 foods were selected to compose the FFQ, in order to avoid the fatigue of the interviewee when filling the instrument. Considering the type of processing and according to the new classification^20^ , the foods were divided into three groups: in natura or minimally processed foods; culinary ingredients and processed foods; and ultra-processed foods. Since the processed culinary ingredients group only had one item (butter), it was added to processed foods.

Defining the level of processing was a complex task, since in the HBS data it is impossible to distinguish whether certain foods are industrialized or not. To minimize the lack of data, the dietary habits of the region were taken into account, considering how the food is most consumed. For example, foods such as lasagna, pizza, sliced bread, hamburger sandwich and flavored yogurts (considered a dairy drink, since, because they have flavorings, they probably contain dyes) were considered industrial preparations. On the other hand, as in the region it is more common to consume filtered coffee and farofa prepared at home, these products, which may have industrialized versions, were considered minimally processed. For preparations, the base food was considered, with space to include information regarding the addition of other foods. In the case of feijoada, for example, the basic food is beans, minimally processed, but information could be included regarding the addition of processed or sausage meats.

We organized the food items in the FFQ according to the meals in which they are consumed daily. For example, foods present in the Northeast region, such as tapioca, chicken egg and coffee with milk, appeared in sequence, to optimize the respondent’s memory. According to literature and considering that cognitive processing is complex, the organization of the list of foods in an FFQ must respect the mental image of meals^21^ .

### Serving Size

The FFQ developed is quantitative, with closed questions about the size of the servings, defined as S, M, L or XL. The individual is asked to indicate their servings consumed in the previous year, considering as a reference the average serving (M). The respondent then selects L if the consumption is less than the reference one; M if it is equal; L if it is greater; and XL if *much bigger* that the reference serving.

To estimate the serving size, the 25th, 50th, 75th and 95th percentiles were used, established for each of the foods on the final list, considering the servings of the two days of the FR of each individual, separately. For grouped foods, the items were considered separately, to estimate the percentiles of the grouping. In the aggregate items (for example, “Other cheeses”), the percentiles were estimated considering the percentiles of the quantities consumed of the types of cheese corresponding to the item (buffalo cheese, reino, minas, canastra, ricotta, provolone, cream cheese).

For some foods, the estimated percentiles (25, 50, 75 and 95) coincided due to the low variation in the size of the servings consumed. In these cases, cross-multiplication was used to estimate only the coincident percentiles. So, for example, in the preparation “baião de dois”, the result found indicated that the 25th and 50th percentiles coincided (75 grams). In this case, the 25th percentile was estimated by cross-multiplication, considering as reference the 50th percentile:

**Figure:**
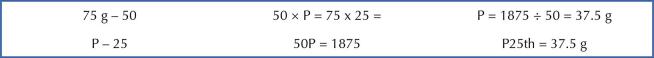

The frequency categories were defined in variation from “never” to 10, and the previous time to estimate the frequency of food consumption was the previous year, covering seasonal variations in food consumption. Due to the amplitude of the consumption frequency adopted, the diagram chosen was the same used by Cardoso and Stocco^22^ in an FFQ for Japanese immigrants.

An initial section provides instructions, developed by a nutritionist, to complete the instrument, with or without the help of an interviewer. At the end of the FFQ, there are seven extra questions that aim to obtain more detailed information about meat meals, such as skin intake, apparent fat and how its prepared (in view of the wide variety of preparations of this food item). The interviewee was also asked about adding salt to meals already prepared, how often they sweeten drinks and type of substance used to sweeten them. The answers to these questions are closed, with the option, for some questions, to tick the answer “Another way” and answer: “Which?”.

### Categorization of the Data of the Studied Population

The study population was described using the variables age and schooling. By age, participants were classified as “young adult” (between 20 and 39 years old) or “adult” (between 40 and 59 years old). As for schooling, the classification was by age groups: up to elementary school (≤ 4 years); up to complete middle school (5 to 8 years of study); incomplete high school (9 to 11 years of study); and complete or incomplete higher education (12 or more years of study).

Income was defined considering the average value of the minimum wage in force at the time of the study (2008–2009): R 440,00. The Body Mass Index was classified according to the criterion of the World Health Organization^b^ . The statistical analyses were descriptive, showing absolute frequency, percentage and 95% confidence intervals.

## RESULTS

The mean age of the sample was 36.5 years (SD = 10.9 years), and 53.4% of the people were female. Most adults were between 20 and 39 years old, approximately 60% had up to eight years of study, 67.4% had per capita income of up to a minimum wage, and 44.1% were overweight or obese ( Table 1 ).

**Table 1:** Characterization of the sample of adults from northeastern Brazil, 2008–2009 HBS.

Variable	Male		Female		Total	

	n	% (95%CI)	n	% (95%CI)	n	% (95%CI)
Age (years)						
Young adult (20 to 39 years)	2,213	63.2 (61.6–64.8)	2,379	59.3 (57.7–60.8)	4,592	61.1 (59.9–62,2)
Adult (40 to 59 years)	1,288	36.8 (35.2–38.4)	1,636	40.7 (39.2–42.3)	2,924	38.9 (37.8–40.0)
Education (years of study)a						
≤ 4	1,463	42.1 (40.5–43.7)	1,409	35.3 (33.9–36.9)	2,872	38.5 (37.4–39.6)
5 to 8	786	22.6 (21.3–24.0)	848	21.3 (20.0–22.6)	1,634	21.9 (20.9–22.9)
9 to 11	972	28.0 (26.5–29.5)	1,282	32.2 (30.7–33.6)	2,254	30.2 (29.2–31.3)
≥ 12	254	7.3 (6.5–8.2)	445	11.2 (10.2–12.2)	699	9.4 (8.7–10.0)
Per capita income (minimum wages)^b^						
< ¼	438	12.5 (11.3–13.4)	469	11.7 (10.7–12.7)	907	12.1 (11.3–12.8)
≥ ¼ < ½	874	25.0 (23.6–26.4)	1,035	25.8 (24.5–27.3)	1,909	25.4 (24.5–26.5)
≥ ½ < 1	1,037	29.6 (28.1–31.1)	1,213	30.2 (28.8–31.7)	2,250	29.9 (28.9–30.9)
≥ 1 < 5	1,038	29.6 (28.3–31.3)	1,186	29.5 (28.1–30.9)	2,224	29.6 (28.6–30.7)
≥ 5	114	3.3 (2.7–3.9)	112	2.8 (2.3–3.4)	226	3.0 (2.7–3.4)
BMI (Kg/m^2^) ^c^						
< 18.5	81	2.3 (1.8–2.9)	177	4.4 (3.8–5.1)	258	3.4 (3.0–3.9)
≥ 18.5 < 25	1,898	54.2 (52.6–55.9)	2,045	50.9 (49.3–52.4)	3.943	52.4 (51.3–53.5)
≥ 25 < 30	1,166	33.3 (31.8–34.9)	1,187	29.6 (28.2–31.0)	2,353	31.3 (30.3–32.4)
≥ 30	356	10.2 (9.2–11.2)	606	15.1 (14.1–16.3)	962	12.8 (12.1–13.6)

^a^ Ignored values for 57 people due to lack of data, representing 0.8% of the sample; ^b^ MW: minimum wage, considering the average value of the MW of the years 2008 and 2009 (R$440,00); ^c^ BMI: Body Mass Index, according to criteria of the World Health Organization (1998).

As already described, after analyzing the completed FR and with the detailed groupings in the methodology finished, we defined a list of foods with 421 items. After the application of the percentage contribution method in the list of energy consumption, macronutrients and micronutrients, 83 of these items remained in the final list of the FFQ, with a contribution of up to 90% of food consumption. Table 2 describes the serving size of food components in the list.

**Table 2:** Food items of the quantitative food frequency questionnaire according to serving size in percentiles (grams) of adults from Northeast Brazil, 2008–2009.

Food	P25	P50	P75	P95
In natura or minimally processed				
Rice	90	125	180	300
Brown rice	126	189	200	351
Baião de dois	165	220	275	380
Banana	37.5^a^	75	150	225
Sweet potato	140	150	300	506
Potato	60	110	165^a^	220
Beiju	62.5^a^	125	187.5^a^	250
Coffee	50	150	240	285^a^
Coffee with milk	150	200	240	300
Bean broth	65^a^	130	260	520
Soup	325	530	780^a^	786.5
Beef with bone (rib, steak, etc.)	70	80	140	300
Beef without bone (top sirloin, picanha, lombo, maminha, etc.)	70	90	105	200
Goat meat	70	140	210	280
Chicken with bone	55	110	165^a^	200
Chicken without bone (fillet, breast)	140	180	200	420
Ground meat or meatball	63	75	120	240
Pork	95	190	285	475
Couscous	72	135	270	405
Cassava flour	23	40	48	123.15
Farofa	15	30	58	135
Beans (black, pinto, kidney, rosinha etc.)	70^a^	140	280	420
Cowpea	105	140	280	420
Green beans	70^a^	140	280	420
Feijoada	112.5^a^	225	450	675
Beef liver	70	100	200	300
Guava	85^a^	170	340	510
Yam	60	90	120	186
Orange (pera, seleta, sweet, da-terra, etc)	90^a^	180	360	540
Whole cow's milk	200	240	360^a^	480
Whole cow's milk powder	16	26.7	32	48
Apple	75^a^	150	225	300
Cassava	105	200	300	400
Papaya	155	170	255^a^	310
Mango	70^a^	140	280	420
Maria-isabel	120	180	240	372
Watermelon	150	200	300^a^	400
Porridge (corn, oats, flour, etc.)	195	220	230	375
Chicken egg	25^a^	50	100	150
Fish (whole, fillet etc.)	100^a^	200	400	600
Popcorn (natural)	10	20^a^	30	40
Salad or raw vegetables, other than fruit	40^a^	80	120^a^	160
Soup (vegetables, meat etc.)	325	520	780^a^	1,040
Fruit juice	120^a^	240	300	480
Tapioca	25^a^	50	75^a^	100
Tomato	30	50	80	100
Fruit smoothie	240	300	450^a^	600
Culinary ingredients and processed foods				
Simple cake without icing	30^a^	60	80	180
Jerky	40	65	130	260
Carne de sol	65	130	195	325
Beer (with or without alcohol)	480	900	1400	3,506
Coxinha	25^a^	50	75^a^	100
Noodles	75	105	150	330
Butter with or without salt	5^a^	10	20	30
Other cheeses (buffalo cheese, reino, minas, canastra, ricotta, provolone, cream cheese)	10	20	40	80
Bread	25^a^	50	100	150
Sweet bread	25^a^	50	100	150
Pastel (cheese, meat, heart of palm etc.)	16^a^	32	46	96
Cheese curd	22.5^a^	45	90	200
Mozzarella cheese	10^a^	20	40	60
Ultra-processed				
Chocolate milk	200	240	360^a^	456
Cookies	20	30	40	200
Sandwich cookie	52	78	200	253.33^b^
Crackers	20	30	35	100
Hot dog	62.5^a^	125	187.5^a^	287.5
Chocolate	28.35	170	255^a^	340
Fruit jam of any taste	48	60	145	301.3
Guava	30^a^	60	90^a^	180
Yogurt of any flavor (industrialized)	100^a^	200	240	400
Lasagna (industrialized)	95^a^	190	475	500
Sausage (pork, beef, chicken, mixed etc.)	30	50	60	120
Instant noodles	160^a^	320	330	436
Margarine with or without salt	5^a^	10	20	32
Mortadella	15	30	45	62
Industrialized sliced bread	25	50	75	100
Pizza (industrialized)	102.5	200	300	400
Juice (artificial)	100^b^	240	300	380^b^
Soda	240	250	300	600
Salami	20	40	60	62
Sausage	31	62	93^a^	124
Cold cut sandwich (cheese, ham, salami, mixed)	45^a^	90	135^a^	180
Hamburger, cheeseburger etc.	62.5^a^	125	187.5^a^	250
Industrialized ice cream of any flavor	80	100	160	240

^a^ Cross multiplication estimated the percentile when there was a coincidence of values between two or more percentiles. For estimation purposes, the 50th percentile was considered as the reference value.

^b^ Cross multiplication estimated the percentile when there was a coincidence of values between two or more percentiles. For estimation purposes, the 75th percentile was considered as the reference value.

^c^ Cross multiplication estimated the percentile when there was a coincidence of values between two or more percentiles. For estimation purposes, the 95th percentile was considered as the reference value.

**Figure 2:**
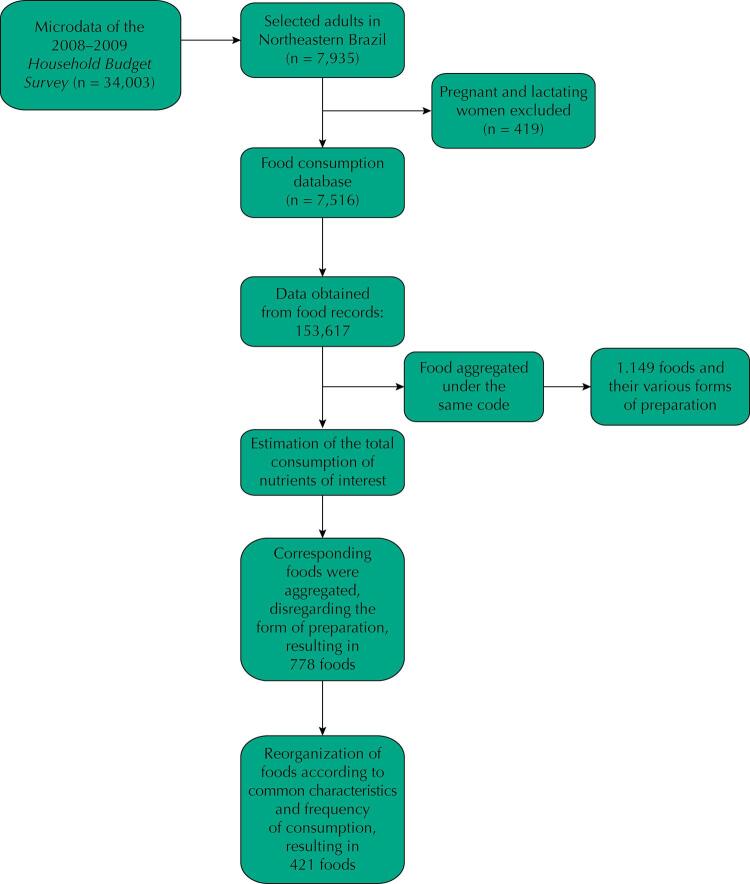
Part of a quantitative food frequency questionnaire built for adults, in a region of Brazil, composed of in natura or minimally processed foods.

Figure 2 shows a suggested presentation format of part of the FFQ, considering food item, frequency of consumption, unit of time and serving size.

## DISCUSSION

It is difficult to record the food intake of an individual, since the measures of food consumption are subjective and there are several variables to consider, such as eating habits, intrapersonal variability, diet complexity, quality of information obtained, age, memory of the respondent, socioeconomic status and exposure factors^1^ . Therefore, methodological rigor is necessary when developing food survey instruments.

Monitoring long-term food consumption trends is important, because with this information one can understand the relation between the dietary factor and diseases^3^ . The type of instrument chosen should consider the specifics of the study and the target population. The developed FFQ can be used, for example, to estimate the usual consumption of NCD-related nutrients among the target population.

The FFQ innovates by considering changes in the habits of Brazilians and categorizing foods by type of processing rather than by food group. The instrument was built specifically for the study population, with the knowledge that diet can be influenced by ethnicity, culture, socioeconomic profile and individual preferences^23^ . The FFQ consists of a list of the main food items that contribute to the target nutrients of the study. This list should be reduced to the maximum by selecting one of the several methods described in literature^1 , 17^ , which range from food identification based on nutrient content and selection with the help of a nutritionist to stepwise multiple analysis^1^ .

In the search to include the most representative foods of the population’s food intake, in our study we opted for the methodology of Block et al.^17^ . The cut-off point for the relative contribution of the item in all lists was 90%, which is recommended by literature. With this cutoff point, the list resulted in 83 food items, therefore below the number of 100 Foods, which should not be exceeded^1^ .

We organized the food items in the FFQ according to the meals in which they are consumed daily. The organization of the list respected the mental image of the meals, since, given the complexity of the cognitive processing, the disposition of the items can help the interviewees to remember the meals consumed in the time interval considered^21^ .

We decided to develop a quantitative instrument, which therefore included the size of the servings. This inclusion is controversial, since the serving consumed by the individual may diverge from the standards established in the questionnaire, which would generate inaccuracy^21^ . However, a case-control study that applied an FFQ directed to the consumption of preformed vitamin A and beta-carotene observed that questions regarding serving size are useful, since they provide additional information on food consumption^24^ .

In order to obtain more detailed data, a range of consumption frequency ranging from 1 to 10 times was established, in addition to the “never” option, as also proposed by Cardoso and Stocco^22^ . The reference period varies according to the study and the target population, but the previous year is most often used for epidemiological purposes^23^ , since diets tend to correlate from one year to another^1^ .

Studies have indicated an increase in the amount of ultra-processed foods, rich in calories, fats, salt and sugar in the diet of Brazilians, while the consumption of in natura or minimally processed foods has been the same, but in a smaller proportion^6 , 25^ . The foods on the list of the developed FFQ confirm this trend in the specific case of Northeastern people, in whose diet there are both minimally processed and traditional foods, such as rice and beans, as well as items such as ice cream, cookies and crackers, and industrialized cakes, classified as ultra-processed.

One study showed that ultra-processed foods increase the energy density of the diet and the consumption of saturated fat, trans fat and sugar, as well as reduces the intake of dietary fiber and micronutrients such as iron, zinc and vitamin A^26^ . Thus, ultra-processed foods are associated with unhealthy dietary nutritional profiles and NCDs^27^ .

Brazil has been adopting strategies to prevent diseases and promote the health and well-being of the population. An example is the current *Guia alimentar para a população brasileira* , which brings information and recommendations on food, meals and eating practices. Based on the NOVA classification, the guide categorizes foods according to degree of processing: in natura or minimally processed, culinary ingredients, processed foods and ultra-processed foods^11^ .

The FFQ shown here seeks to collect food consumption data that allow to evaluate adherence to the recommendations of the *Guia alimentar para a população brasileira* . The choice to consider the type of processing comes precisely from this new emphasis, because the conventional classification of foods, according to nutrients, often groups in the same category items with very different effects on health^12^ .

In this study, we joined the categories “culinary ingredients” and “processed foods”, since the only culinary ingredient that entered the list was butter. As culinary ingredients consist of processed foods^12^ , we decided to put the two categories together. The first two pages of the FFQ show guidelines for completing the instrument, and a final section brings extra questions. Instruments of this type can include this section in order to collect data on cooking form of food, consumption of fats and condiments, addition of salt and even brand of products consumed^28^ .

The study assumes the limitations arising from the original research, such as the use of FR, an instrument that can generate some inaccuracy, since the individual has knowledge of what is being evaluated. Furthermore, the food record, because it is filled in by the participants themselves, excludes illiterate individuals. On the other hand, it is worth highlighting the advantage of minimizing memory bias, since the record is made at the time of consumption^1^ . The table of nutritional composition of the foods used was compiled from the HBS itself, which has limitations such as lack of data on nutrients for some foods and repetition of identical data for foods with different forms of preparation, which makes it difficult to classify foods according to processing level. Another limitation was the impossibility of evaluating free sugars also due to the lack of data. Sugar from processed and ultra-processed foods, however, has been replacing table sugar as the main source of sugar consumption in recent decades^29^ .

The development of FFQ for the North and Northeast regions of the country is still minimal when compared to the other regions, and the questionnaires developed so far are directed to specific states or cities^21 , 29^ . To date, there is no knowledge of an instrument developed to evaluate the food consumption of adults throughout the Northeast region of the country. Thus, the FFQ shown here stands out as both original and relevant, since its design allows to discriminate differences in feeding between populations^30^ .

Because it is a cheap instrument, widely used in large epidemiological studies and able to estimate the usual consumption in a given period of time^1^ , the FFQ can be used to capture changes in Brazilian food consumption^5^ . In addition, the instrument innovates by classifying foods according to the level of processing. Given the increasing consumption of processed and ultra-processed products, the proposal shown here can serve as a model to develop other questionnaires, including for other regions of Brazil or even other countries, since the trend of industrialized food consumption is global.

Finally, it should be noted that the FFQ developed must still undergo a pilot test to verify the coherence of the questions and the time of application, followed then by validation and reproducibility processes, after which it can be used in epidemiological studies with the target population.

## Footnotes

a Pontifícia Universidade Católica do Rio de Janeiro. DataZoom: simplifying access to Brazil’s microdata [Internet]. Available from: http://www.econ.pucrio.br/datazoom/index.html9

b World Health Organization. Physical status: the use and interpretation of anthropometry. Report of a WHO Expert Committee. World Health Organ Tech Rep Ser [Internet]. 1995;854:1–452. Available from: http://www.ncbi.nlm.nih.gov/pubmed/8594834
