# Supplementary material for: Food frequency questionnaire for adults in the Brazilian Northeast region: emphasis on the level of food processing
Source: Rev Saude Publica. 2021 Aug 6;55:51. doi: 10.11606/s1518-8787.2021055002473 (PMC8352563; doi:10.11606/s1518-8787.2021055002473)
Supplement: Translation

# Translation: Questionário de frequência alimentar para adultos da região Nordeste: ênfase no nível de processamento dos alimentos

**Authors:** Virginia Williane de Lima Motta, Severina Carla Vieira Cunha Lima, Dirce Maria Lobo Marchioni, Clélia de Oliveira Lyra

**Affiliations:** I Brasil Universidade Federal do Rio Grande do Norte. Centro de Ciências da Saúde. Programa de Pós-Graduação em Nutrição. Natal, RN, Brasil; II Brasil Universidade Federal do Rio Grande do Norte. Centro de Ciências da Saúde. Departamento de Nutrição. Natal, RN, Brasil; III Brasil Universidade de São Paulo. Faculdade de Saúde Pública. Departamento de Nutrição. São Paulo, SP, Brasil

**Keywords:** Adulto, Consumo de Alimentos, Alimentos Industrializados, Inquéritos e Questionários, Doenças não transmissíveis, prevenção & controle

## Abstract

**OBJETIVO:**

Desenvolver um Questionário de Frequência Alimentar (QFA) quantitativo para adultos da região Nordeste do Brasil, com o fim de identificar a frequência de consumo de alimentos considerados de proteção e risco para doenças crônicas não transmissíveis (DCNT), agrupando os itens alimentares por nível de processamento.

**MÉTODOS:**

Para desenvolver o QFA foram utilizados dados de 7.516 adultos do Nordeste do Brasil, extraídos da Pesquisa de Orçamentos Familiares 2008–2009. As listas de alimentos foram elaboradas segundo a metodologia da contribuição relativa do item, nas quais foram identificados os itens alimentares com maior contribuição relativa para macronutrientes, fibra, gordura saturada, gordura trans, sódio e potássio. Tais listas foram compostas de todos os alimentos cujo somatório de contribuição foi de até 90%. Na estrutura final do QFA, os itens alimentares foram organizados de modo a respeitar a imagem mental das refeições.

**RESULTADOS:**

O QFA resultou em 83 itens alimentares, distribuídos em minimamente processados, processados e ultraprocessados. O ano anterior foi escolhido como tempo para estimar o consumo dos alimentos, e as opções de frequência variaram de “nunca” até “10 vezes”. O instrumento inclui orientações para preenchimento e colhe dados sobre o tamanho das porções (pequena, média, grande e extragrande), bem como informações complementares sobre as preparações culinárias. Registrou-se um percentual elevado de pessoas com excesso de peso (44,1%).

**CONCLUSÃO:**

O estudo culminou em um QFA para identificar a frequência de consumo de alimentos considerados de proteção e risco para DCNT. O instrumento pode subsidiar estudos epidemiológicos que avaliem desfechos relacionados à dieta de adultos considerando o nível de processamento de alimentos, em consonância com o *Guia alimentar para a população brasileira* .

## INTRODUÇÃO

O Questionário de Frequência Alimentar (QFA) é um método de inquérito alimentar utilizado em investigações epidemiológicas para coletar informações sobre consumo alimentar e dietético. Seu objetivo é investigar a relação entre dieta e doença^1^ .

O QFA, cujo precursor é o *checklist* desenvolvido por Burke^2^ , tem entre suas vantagens: capacidade de avaliar a dieta habitual sem alterar o padrão de consumo alimentar, baixo custo e tempo menor de preenchimento em comparação ao registro alimentar^1^ . A limitação do instrumento, por outro lado, está no fato dele documentar a ingestão alimentar dos indivíduos dentro de um determinado período de tempo, o que pode levar a relatos distorcidos pelo viés de memória, apresenta também uma baixa precisão ao quantificar a dieta^3^ .

Para minimizar as limitações de um QFA é necessário seguir com rigor a metodologia apropriada, a fim de se obter instrumentos precisos e economicamente viáveis. Conforme o objetivo do estudo, o pesquisador deve sistematizar os itens que compõem o questionário, como lista de alimentos, categorias de frequência de consumo e tipo de questionário: qualitativo, semiquantitativo ou quantitativo^4^ . O tipo qualitativo não inclui tamanho das porções, enquanto o semiquantitativo inclui. Já o quantitativo inclui tamanhos de referência: pequena, média e grande^1^ .

No Brasil, a dieta tradicional é marcada pela ingestão de alimentos como arroz, feijão e frutas. Tem-se observado, no entanto, modificações nessa dieta, em todas as faixas etárias^5^ . Alimentos *in natura* ou minimamente processados vêm sendo substituídos por alimentos ultraprocessados^6^ .

Estas alterações na dieta tradicional brasileira são acompanhadas pelo aumento da prevalência do diabetes, da obesidade e demais doenças crônicas não transmissíveis (DCNT). Uma dieta inadequada, com alto consumo de ultraprocessados (produtos ricos em sal, gorduras saturadas, gorduras trans e açúcares)^7^ , é um dos fatores de risco modificáveis relacionados a DCNT em todo o mundo^8^ .

Apesar de a região Nordeste, aqui estudada, destacar-se por sua culinária característica, com alimentos regionais considerados boas fontes de diversos nutrientes^9^ , a população nordestina tem apresentado elevadas prevalências de DCNT^10^ . Por isso a importância de se desenvolver um questionário específico para a região, capaz de acompanhar a relação entre consumo alimentar e processo saúde-doença com foco no nível de processamento de alimentos.

O Guia alimentar para a população brasileira^11^ direciona as recomendações de consumo de alimentos de acordo com a classificação NOVA, neste âmbito o QFA poderia ser utilizado em estudos epidemiológicos com o propósito de avaliar a adesão das recomendações pela população. No entanto, ainda são limitados os estudos que buscam estabelecer a relação entre consumo de alimentos ultraprocessados e saúde, visto que não há instrumentos específicos para avaliar o consumo desses produtos, e os instrumentos tradicionais não foram desenvolvidos com esse propósito^12^ .

Assim, o objetivo deste estudo foi desenvolver um QFA quantitativo para adultos da região Nordeste do Brasil, com o fim de identificar a frequência de consumo de alimentos considerados de proteção e risco para DCNT. No instrumento, os itens alimentares foram agrupados por nível de processamento.

## MÉTODOS

O presente estudo utiliza dados de consumo alimentar pessoal do Inquérito Nacional de Alimentação (INA) 2008–2009, um módulo da Pesquisa de Orçamentos Familiares (POF) de que participaram 34.003 indivíduos com 10 anos ou mais de idade. Por dois dias consecutivos, os participantes preencheram registros alimentares (RA) em que anotavam horário e local do consumo de alimento, quantidades em medidas caseiras e forma de preparo. Os demais detalhes sobre amostragem e coleta de dados da POF encontram-se publicados em documento oficial da pesquisa^5^ .

Para compor a amostra deste estudo, foram consideradas as 7.516 pessoas da região Nordeste, entre 20 e 59 anos de idade, que preencheram os registros alimentares na POF. Gestantes e lactantes (n = 419) não foram incluídas. Como o estudo utilizou banco de dados secundário, de domínio público, a submissão a Comitê de Ética em Pesquisa não foi necessária.

### Construção do Banco de Dados e Análise

Os dados da pesquisa foram obtidos no Sistema IBGE de Recuperação Automática (Sidra), por meio de *download* de microdados com informações codificadas de todos os moradores dos domicílios que participaram da POF 2008–2009. Para importar e ler os dados, foi utilizado o pacote estatístico Data Zoom, versão Stata 12 para Windows^a^ .

Para obter a lista de alimentos foram acessados dois bancos de dados: um referente às características dos indivíduos (REGISTRO: PESSOAS - POF1) e outro com informações sobre o consumo alimentar individual (REGISTRO: CONSUMO ALIMENTAR - POF7). As quantidades consumidas dos alimentos foram transformadas em gramas ou mililitros, tomando como base a Tabela de Medidas Referidas para os Alimentos Consumidos no Brasil da POF 2008–2009^13^ .

As informações obtidas pela soma dos registros alimentares da amostra representaram 153.617 dados alimentares, ou seja, todos os alimentos consumidos pela amostra nos dois dias de registro. Optou-se por somar os alimentos registrados nos dois dias com o objetivo de incluir o maior número possível de alimentos mais frequentemente consumidos pela população em questão. Foram identificados, então, 1.149 alimentos, em suas diversas formas de preparo (por exemplo, “frango cozido” e “frango assado”).

O consumo de energia, macronutrientes, fibra, gordura saturada, gordura trans, sódio e potássio foi calculado a partir da Tabela de Composição Nutricional dos Alimentos Consumidos no Brasil da POF^14^ . Os nutrientes foram escolhidos considerando características dos alimentos relacionadas a proteção ou risco para DCNT^15 , 16^ . Além dos nutrientes citados anteriormente, um componente considerado crítico para DCNT são os açúcares livres^15 , 16^ . Nos dados da POF, no entanto, não constam informações sobre esse componente. Não sendo possível sua avaliação.

Na etapa seguinte, atribuiu-se um código por alimento, independentemente das formas de preparo, com exceção das carnes fritas, que permaneceram separadas. Apesar das limitações da Tabela de Composição Nutricional, especialmente quanto à falta de informações sobre gordura trans de acordo com o preparo, optou-se por manter as preparações fritas separadas, dada a diferença no teor de gordura.

Posteriormente, foram somados os valores dos nutrientes de interesse de alimentos com códigos iguais. Por exemplo: as calorias do frango cozido foram somadas às calorias do frango assado, e ambas codificadas como frango. Ao final desse processo, os 1.149 alimentos foram reduzidos a 778 alimentos.

Alimentos que não apresentavam descrição específica (p. ex., refrigerante não especificado), ou apresentavam descrição similar à de outro alimento (p. ex.: carne moída e almôndega), foram incluídos em um único item, por equivalência. Identificou-se também a necessidade de agrupar alimentos pouco consumidos (citados menos de 20 vezes^17^ ) em um só item, considerando a similaridade entre eles. Assim, foram criados três novos grupos: “Outras frutas”, “Outros queijos” e “Outras bebidas alcoólicas”. Em relação às carnes de boi e de frango, estas foram agregadas segundo a característica “com osso” e “sem osso”, dada a diferença na quantidade de gordura. Após todos os ajustes, 421 alimentos foram considerados para a lista do QFA ( Figura 1 ).

**Figura 1:** Fluxograma da organização do banco de dados para construção de um Questionário de Frequência Alimentar.

N: nunca; D: dia; S: semana; M: mês; A: ano; P: pequeno; M: médio; G: grande; EG: extragrande.

### Lista de Alimentos

Para construir as listas de alimentos com base nos nutrientes de interesse, optou-se pela metodologia de Block et al., que considera a contribuição relativa do item^17^ , identificando os itens com maior contribuição relativa para macronutrientes, fibra, gordura saturada, gordura trans, sódio e potássio. As listas foram compostas por todos os alimentos cuja soma de contribuição foi de até 90%, como estabelecido em outros estudos^18 , 19^ .

Neste estudo não foram selecionados mais do que 100 alimentos para compor o QFA, visando evitar a fadiga do entrevistado ao preencher o instrumento. Considerando o tipo de processamento e de acordo com a Classificação NOVA^20^ , os alimentos foram divididos em três grupos: alimentos *in natura* ou minimamente processados; ingredientes culinários e alimentos processados; e alimentos ultraprocessados. Os ingredientes culinários processados foram unidos aos alimentos processados, pois havia apenas um alimento no grupo dos ingredientes culinários: a manteiga.

Definir o nível de processamento foi uma tarefa complexa, visto que nos dados da POF não é possível distinguir se certos alimentos são industrializados ou não. Para minimizar a falta de dados, levou-se em conta os hábitos alimentares da região, considerando a forma como o alimento é mais consumido. Por exemplo, foram consideradas preparações industriais alimentos como lasanha, pizza, pão de forma, sanduíche tipo hambúrguer e iogurtes com sabor (considerados uma bebida láctea, já que, por terem sabor, provavelmente contêm corantes). Por outro lado, como na região é mais habitual que se consuma café filtrado e farofa preparada em domicílio, esses produtos, que podem ter versões industrializadas, foram considerados minimamente processados. Para preparações, considerou-se o alimento-base, com espaço para incluir informação referente à adição de outros alimentos. No caso da feijoada, por exemplo, o alimento-base é o feijão, minimamente processado, mas poderiam ser incluídas informações referentes à adição de carnes processadas ou embutidos.

Os itens alimentares foram organizados no QFA de acordo com as refeições em que são consumidos diariamente. Por exemplo, alimentos presentes no desjejum da região Nordeste, como tapioca, ovo de galinha e café com leite, apareceram em sequência, para otimizar a memória do respondente. De acordo com a literatura e considerando que o processamento cognitivo é complexo, a organização da lista dos alimentos em um QFA deve respeitar a imagem mental das refeições^21^ .

### Tamanho das Porções

O QFA desenvolvido é de tipo quantitativo, com perguntas fechadas sobre o tamanho das porções, definidas como P, M, G ou EG. Pede-se ao indivíduo que indique sua porção de consumo no ano anterior, considerando como referência a porção média (M). O respondente então seleciona P caso o consumo seja menor que o de referência; M se for igual; G se for maior; e EG se *bem maior* que a porção de referência.

Para calcular o tamanho das porções foram utilizados os percentis 25, 50, 75 e 95, estabelecidos para cada um dos alimentos da lista final, considerando as porções dos dois dias do RA de cada indivíduo, separadamente. Para os alimentos agrupados, os itens foram considerados separadamente, para calcular os percentis do agrupamento. Nos itens agregados (por exemplo, “Outros queijos”), os percentis foram calculados considerando os percentis das quantidades consumidas dos tipos de queijo correspondentes ao item (búfala, reino, minas, canastra, ricota, provolone, *cream cheese* ).

Para alguns alimentos, os percentis calculados (25, 50, 75 e 95) coincidiram devido à baixa variação no tamanho das porções consumidas. Nestes casos, utilizou-se a regra de três para calcular apenas os percentis coincidentes. Assim, por exemplo, na preparação “baião de dois”, o resultado encontrado apontava que os percentis 25 e 50 eram coincidentes (75 gramas). Neste caso, o percentil 25 foi calculado por regra de três, considerando como referência o percentil 50:

As categorias de frequências foram definidas em variação de “nunca” a 10, e o tempo pregresso para estimar a frequência de consumo dos alimentos foi o ano anterior, abrangendo as variações sazonais no consumo alimentar. Devido à amplitude da frequência de consumo adotada, a diagramação escolhida foi a mesma utilizada por Cardoso e Stocco^22^ em um QFA para imigrantes japoneses.

Uma seção inicial traz instruções, desenvolvidas por nutricionista, para preencher o instrumento, com ou sem auxílio de entrevistador. Ao final do QFA, há sete perguntas extras que visam obter informações mais detalhadas sobre refeições cárneas, como ingestão de pele e gordura aparente e forma de preparo (tendo em vista a grande variedade de preparações desse item alimentar). O entrevistado é questionado, ainda, sobre adição de sal nas refeições já preparadas, frequência com que adoça as bebidas e tipo de substância utilizada para adoçá-las. As respostas a estas questões são fechadas, com a opção, para algumas perguntas, de assinalar a resposta “Outra forma” e responder: “Qual?”.

### Categorização dos Dados da População Estudada

A população de estudo foi descrita por meio das variáveis idade e escolaridade. Em idade, os participantes foram classificados como “jovem adulto” (entre 20 e 39 anos de idade) ou “adulto” (entre 40 e 59 anos). Quanto à escolaridade, a classificação se deu por faixas de anos de estudo: até o 1º ciclo do ensino fundamental (≤ 4 anos); 2º ciclo do fundamental completo (5 a 8 anos de estudo); ensino médio incompleto (9 a 11 anos de estudo); e superior completo ou incompleto (12 ou mais anos de estudo).

A renda foi definida considerando o valor médio do salário mínimo vigente à época do estudo (2008-2009): R$440,00. O Índice de Massa Corporal foi classificado segundo o critério da Organização Mundial da Saúde^b^ . As análises estatísticas foram descritivas, apresentando frequência absoluta, percentual e intervalos de confiança de 95%.

## RESULTADOS

A média de idade da amostra foi de 36,5 anos (DP = 10,9 anos), e 53,4% das pessoas eram do sexo feminino. A maioria dos adultos tinha entre 20 e 39 anos de idade, aproximadamente 60% tinham até oito anos de estudo, 67,4% contavam com renda per capita de até um salário mínimo, e 44,1% apresentavam sobrepeso ou obesidade ( Tabela 1 ).

**Tabela 1:** Caracterização da amostra de adultos do Nordeste brasileiro, POF 2008–2009.

Variável	Masculino		Feminino		Total	
		
	n	% (IC95%)	n	% (IC95%)	n	% (IC95%)
Idade (anos)						
Jovem adulto (20 a 39 anos)	2.213	63,2 (61,6–64,8)	2.379	59,3 (57,7–60,8)	4.592	61,1 (59,9–62,2)
Adulto (40 a 59 anos)	1.288	36,8 (35,2–38,4)	1.636	40,7 (39,2–42,3)	2.924	38,9 (37,8–40,0)
Escolaridade (anos de estudo)^a^						
≤ 4	1.463	42,1 (40,5–43,7)	1.409	35,3 (33,9–36,9)	2.872	38,5 (37,4–39,6)
5 a 8	786	22,6 (21,3–24,0)	848	21,3 (20,0–22,6)	1.634	21,9 (20,9–22,9)
9 a 11	972	28,0 (26,5–29,5)	1282	32,2 (30,7–33,6)	2.254	30,2 (29,2–31,3)
≥ 12	254	7,3 (6,5–8,2)	445	11,2 (10,2–12,2)	699	9,4 (8,7–10,0)
Renda per capita (salários mínimos)^b^						
< ¼	438	12,5 (11,3–13,4)	469	11,7 (10,7–12,7)	907	12,1 (11,3–12,8)
≥ ¼ < ½	874	25,0 (23,6–26,4)	1035	25,8 (24,5–27,3)	1.909	25,4 (24,5–26,5)
≥ ½ < 1	1.037	29,6 (28,1–31,1)	1213	30,2 (28,8–31,7)	2.250	29,9 (28,9–30,9)
≥ 1 < 5	1.038	29,6 (28,3–31,3)	1186	29,5 (28,1–30,9)	2.224	29,6 (28,6–30,7)
≥ 5	114	3,3 (2,7–3,9)	112	2,8 (2,3–3,4)	226	3,0 (2,7–3,4)
IMC (Kg/m^2^)^c^						
< 18,5	81	2,3 (1,8–2,9)	177	4,4 (3,8–5,1)	258	3,4 (3,0–3,9)
≥ 18,5 < 25	1.898	54,2 (52,6–55,9)	2.045	50,9 (49,3–52,4)	3.943	52,4 (51,3–53,5)
≥ 25 < 30	1.166	33,3 (31,8–34,9)	1.187	29,6 (28,2–31,0)	2.353	31,3 (30,3–32,4)
≥ 30	356	10,2 (9,2–11,2)	606	15,1 (14,1–16,3)	962	12,8 (12,1–13,6)

^a^Valores ignorados para 57 pessoas por ausência de dados, representando 0,8% da amostra;^b^SM: salário mínimo, considerando o valor médio do SM dos anos de 2008 e 2009 (R$440,00);^c^IMC: Índice de Massa Corporal, segundo critérios da Organização Mundial da Saúde (1998).

Como já descrito, após análise dos RA preenchidos e feitos os agrupamentos detalhados na metodologia, definiu-se uma lista de alimentos com 421 itens. Depois da aplicação do método de contribuição percentual na lista do consumo de energia, macronutrientes e micronutrientes, restaram 83 desses itens na lista final do QFA, com contribuição de até 90% do consumo alimentar. O tamanho das porções de alimentos componentes da lista se encontra descrito na Tabela 2 .

**Tabela 2:** Itens alimentares do Questionário de Frequência alimentar quantitativo segundo o tamanho das porções em percentis (gramas) de adultos do Nordeste, Brasil, 2008−2009.

Alimento	P25	P50	P75	P95
*In natura* ou minimamente processados				
Arroz	90	125	180	300
Arroz integral	126	189	200	351
Baião de dois	165	220	275	380
Banana	37,5^a^	75	150	225
Batata-doce	140	150	300	506
Batata-inglesa	60	110	165^a^	220
Beiju	62,5^a^	125	187,5^a^	250
Café	50	150	240	285^a^
Café com leite	150	200	240	300
Caldo de feijão	65^a^	130	260	520
Canja	325	530	780^a^	786,5
Carne bovina com osso (costela, bisteca etc.)	70	80	140	300
Carne bovina sem osso (alcatra, picanha, lombo, maminha etc.)	70	90	105	200
Carne caprina	70	140	210	280
Carne de frango ou galinha com osso	55	110	165^a^	200
Carne de frango ou galinha sem osso (filé, peito)	140	180	200	420
Carne moída ou almôndega	63	75	120	240
Carne suína	95	190	285	475
Cuscuz	72	135	270	405
Farinha de mandioca	23	40	48	123,15
Farofa	15	30	58	135
Feijão (preto, mulatinho, roxo, rosinha etc.)	70^a^	140	280	420
Feijão-de-corda	105	140	280	420
Feijão-verde	70^a^	140	280	420
Feijoada	112,5^a^	225	450	675
Fígado bovino	70	100	200	300
Goiaba	85^a^	170	340	510
Inhame	60	90	120	186
Laranja (pera, seleta, lima, da-terra, etc)	90^a^	180	360	540
Leite de vaca integral	200	240	360^a^	480
Leite de vaca em pó integral	16	26,7	32	48
Maçã	75^a^	150	225	300
Macaxeira	105	200	300	400
Mamão	155	170	255^a^	310
Manga	70^a^	140	280	420
Maria-isabel	120	180	240	372
Melancia	150	200	300^a^	400
Mingau (fubá, aveia, farinha etc.)	195	220	230	375
Ovo de galinha	25^a^	50	100	150
Peixe (inteiro, em posta, em filé etc.)	100^a^	200	400	600
Pipoca de milho (natural)	10	20^a^	30	40
Salada ou verdura crua, exceto de fruta	40^a^	80	120^a^	160
Sopa (legumes, carne etc.)	325	520	780^a^	1040
Suco de fruta	120^a^	240	300	480
Tapioca de goma	25^a^	50	75^a^	100
Tomate	30	50	80	100
Vitamina de fruta	240	300	450^a^	600
Ingredientes culinários e alimentos processados				
Bolo simples e sem cobertura	30^a^	60	80	180
Carne de charque	40	65	130	260
Carne de sol	65	130	195	325
Cerveja (com ou sem álcool)	480	900	1400	3506
Coxinha	25^a^	50	75^a^	100
Macarrão	75	105	150	330
Manteiga com ou sem sal	5^a^	10	20	30
Outros queijos (búfala, reino, minas, canastra, ricota, provolone, *cream cheese* )	10	20	40	80
Pão francês	25^a^	50	100	150
Pão doce	25^a^	50	100	150
Pastel (queijo, carne, palmito etc.)	16^a^	32	46	96
Queijo de coalho	22,5^a^	45	90	200
Queijo muçarela	10^a^	20	40	60
Ultraprocessados				
Bebida achocolatada	200	240	360^a^	456
Biscoito doce	20	30	40	200
Biscoito recheado	52	78	200	253,33^b^
Biscoito salgado	20	30	35	100
Cachorro-quente	62,5^a^	125	187,5^a^	287,5
Chocolate	28,35	170	255^a^	340
Doce de frutas de qualquer sabor	48	60	145	301,3
Goiabada	30^a^	60	90^a^	180
Iogurte de qualquer sabor (industrializado)	100^a^	200	240	400
Lasanha (industrializada)	95^a^	190	475	500
Linguiça (suína, bovina, frango, mista etc.)	30	50	60	120
Macarrão instantâneo	160^a^	320	330	436
Margarina com ou sem sal	5^a^	10	20	32
Mortadela	15	30	45	62
Pão de forma industrializado	25	50	75	100
Pizza (industrializada)	102,5	200	300	400
Refresco (artificial)	100^b^	240	300	380^b^
Refrigerante	240	250	300	600
Salame	20	40	60	62
Salsicha	31	62	93^a^	124
Sanduíche de frios (queijo, presunto, salame, misto)	45^a^	90	135^a^	180
Sanduíche tipo hambúrguer, cheesebúrguer etc.	62,5^a^	125	187,5^a^	250
Sorvete de qualquer sabor industrializado	80	100	160	240

^a^O percentil foi calculado por regra de três quando houve coincidência de valores entre dois ou mais percentis. Para efeito de cálculo, considerou-se como valor de referência o percentil 50.

^b^O percentil foi calculado por regra de três quando houve coincidência de valores entre dois ou mais percentis. Para efeito de cálculo, considerou-se como valor de referência o percentil 75.

^c^O percentil foi calculado por regra de três quando houve coincidência de valores entre dois ou mais percentis. Para efeito de cálculo, considerou-se como valor de referência o percentil 95.

Na Figura 2 , sugere-se um formato de apresentação de parte do QFA, considerando item alimentar, frequência de consumo, unidade de tempo e tamanho da porção.

**Figura 2:** Parte de um questionário de frequência alimentar quantitativo construído para adultos, em uma região do Brasil, composta de alimentos *in natura* ou minimamente processados.

## DISCUSSÃO

É difícil registrar a ingestão alimentar de um indivíduo, visto que as medidas de consumo alimentar são subjetivas e há diversas variáveis a considerar, como hábitos alimentares, variabilidade intrapessoal, complexidade da dieta, qualidade da informação obtida, idade, memória do entrevistado, status socioeconômico e fatores de exposição^1^ . Por isso é necessário rigor metodológico ao se desenvolver instrumentos de inquérito alimentar.

Acompanhar as tendências de consumo alimentar a longo prazo é importante, pois com essa informação se pode compreender a relação do fator dietético com as doenças^3^ . O tipo de instrumento escolhido deve considerar as especificidades do estudo e da população-alvo. O QFA desenvolvido pode ser utilizado, por exemplo, para estimar o consumo habitual dos nutrientes relacionados às DCNT entre a população-alvo.

O QFA inova ao considerar as modificações nos hábitos dos brasileiros e categorizar os alimentos por tipo de processamento ao invés de grupo alimentar. O instrumento foi construído especificamente para a população do estudo, com a ciência de que a dieta pode ser influenciada por etnia, cultura, perfil socioeconômico e preferências dos indivíduos^23^ . O QFA é constituído de uma lista com os principais itens alimentares que contribuem com os nutrientes-alvo do estudo. Essa lista deve ser reduzida ao máximo, devendo selecionar um dos diversos métodos descritos na literatura^1 , 17^ , que variam desde identificação de alimentos com base no conteúdo do nutriente e seleção com a ajuda de um nutricionista até análise múltipla *stepwise* ^1^ .

Na busca por incluir os alimentos mais representativos da ingestão alimentar da população, no presente estudo optou-se pela metodologia de Block et al.^17^ . O ponto de corte para a contribuição relativa do item, em todas as listas, foi de 90%, o recomendado pela literatura. Com esse ponto de corte, a lista resultou em 83 itens alimentares, abaixo portanto do número de 100 alimentos, que não é recomendado ultrapassar^1^ .

Os itens alimentares foram organizados no QFA de acordo com as refeições em que são consumidos diariamente. A organização da lista respeitou a imagem mental das refeições, visto que, dada a complexidade do processamento cognitivo, a disposição dos itens pode ajudar os entrevistados a recordar as refeições consumidas no intervalo de tempo considerado^21^ .

Optou-se por desenvolver um instrumento quantitativo, que incluísse, portanto, o tamanho das porções. Essa inclusão é controversa, uma vez que a porção consumida pelo indivíduo pode divergir dos padrões estabelecidos no questionário, o que geraria imprecisão^21^ . Porém, em estudo de caso-controle que aplicou um QFA direcionado ao consumo de vitamina A pré-formada e betacaroteno, observou-se que questões referentes ao tamanho das porções são úteis, pois fornecem informações adicionais sobre o consumo alimentar^24^ .

A fim de obter dados mais detalhados, foi estabelecida uma amplitude de frequência de consumo variando de 1 a 10 vezes, além da opção “nunca”, conforme proposto também por Cardoso e Stocco^22^ . O período de referência varia de acordo com o estudo e a população-alvo, mas o ano anterior é mais frequentemente utilizado para propósitos epidemiológicos^23^ , visto que dietas tendem a se correlacionar de um ano para outro^1^ .

Estudos têm apontado aumento da quantidade de alimentos ultraprocessados, ricos em calorias, gorduras, sal e açúcar na dieta dos brasileiros, enquanto o consumo de alimentos *in natura* ou minimamente processados tem se mantido, mas em menor proporção^6 , 25^ . Os alimentos da lista do QFA desenvolvido confirmam essa tendência no caso específico dos nordestinos, em cuja dieta há tanto alimentos minimamente processados e tradicionais, como arroz e feijão, quanto itens como sorvete, biscoitos e bolos industrializados, classificados como ultraprocesssados.

Um estudo mostrou que alimentos ultraprocessados aumentam a densidade energética da dieta e o consumo de gordura saturada, gordura trans e açúcar, além de reduzir a ingestão de fibra dietética e micronutrientes como ferro, zinco e vitamina A^26^ . Assim, os ultraprocessados estão associados a perfis nutricionais dietéticos não saudáveis e DCNT^27^ .

No Brasil estratégias têm sido adotadas, para prevenir doenças e promover a saúde e o bem-estar da população. Um exemplo é o atual *Guia alimentar para a população brasileira* , que traz informações e recomendações sobre alimentos, refeições e práticas alimentares. Com base na Classificação NOVA, o guia categoriza os alimentos de acordo com grau de processamento: *in natura* ou minimamente processados, ingredientes culinários, alimentos processados e alimentos ultraprocessados^11^ .

O QFA aqui apresentado busca coletar dados de consumo alimentar que permitam avaliar a adesão às recomendações do *Guia alimentar para a população brasileira* . A escolha por considerar o tipo de processamento advém justamente dessa nova ênfase, pois a classificação convencional dos alimentos, de acordo com nutrientes, muitas vezes agrupa numa mesma categoria itens com efeitos muito diferentes sobre a saúde^12^ .

No presente estudo, as categorias “ingredientes culinários” e “alimentos processados” foram unidas, pois o único ingrediente culinário que entrou na lista foi a manteiga. Como ingredientes culinários consistem em alimentos processados^12^ , as duas categorias foram unidas. As duas primeiras páginas do QFA apresentam orientações para o preenchimento do instrumento, e uma seção final traz perguntas extras. Instrumentos desse tipo podem incluir essa seção a fim de colher dados sobre forma de cocção dos alimentos, consumo de gorduras e condimentos, adição de sal e até mesmo marca dos produtos consumidos^28^ .

O estudo assume as limitações advindas da pesquisa original, como uso do RA, instrumento que pode gerar certa imprecisão, visto que o indivíduo tem conhecimento de que está sendo avaliado. Ademais, o registro alimentar, por ser preenchido pelo próprio participante, exclui indivíduos não alfabetizados. Por outro lado, cabe destacar a vantagem da minimização do viés de memória, uma vez que o registro é feito no momento do consumo^1^ . A tabela de composição nutricional dos alimentos utilizada foi o compilado da própria POF, que apresenta limitações como falta de dados sobre nutrientes para alguns alimentos e repetição de dados idênticos para alimentos com diferentes formas de preparo, o que dificulta a classificação dos alimentos segundo nível de processamento. Uma outra limitação foi a impossibilidade de avaliar os açúcares livres também por conta da falta de dados. O açúcar proveniente de alimentos processados e ultraprocessados, no entanto, vem substituindo o açúcar de mesa como principal fonte de consumo de açúcares nas últimas décadas^29^ .

O desenvolvimento de QFA para as regiões Norte e Nordeste do país ainda é ínfimo quando comparado às demais regiões, e os questionários desenvolvidos até o momento são direcionados a estados ou cidades específicas^21 , 29^ . Até o presente momento, não se tem conhecimento de um instrumento elaborado para avaliar o consumo alimentar de adultos de toda a região Nordeste do país. Assim, o QFA aqui apresentado destaca-se tanto como original quanto relevante, visto que seu desenho permite discriminar diferenças na alimentação entre populações^30^ .

Por ser um instrumento barato, bastante utilizado em grandes estudos epidemiológicos e capaz de estimar o consumo habitual em um dado período de tempo^1^ , o QFA pode ser utilizado para captar as mudanças no consumo alimentar dos brasileiros^5^ . Ademais, o instrumento inova ao classificar os alimentos de acordo com o nível de processamento. Dado o crescente consumo de processados e ultraprocessados, a proposta aqui apresentada pode servir de modelo para desenvolver outros questionários, inclusive para outras regiões do Brasil ou até outros países, visto que a tendência de consumo alimentar de industrializados é global.

Por fim, destaca-se que o QFA desenvolvido ainda deve passar por um teste-piloto para verificar a coerência das perguntas e o tempo de aplicação, seguindo posteriormente para o processo de validação e reprodutibilidade, após o qual poderá ser utilizado em estudos epidemiológicos com a população-alvo.

## Footnotes

a Pontifícia Universidade Católica do Rio de Janeiro. DataZoom: simplificando o acesso aos microdados do Brasil [Internet]. Disponível em: http://www.econ.pucrio.br/datazoom/index.html9

b World Health Organization. Physical status: the use and interpretation of anthropometry. Report of a WHO Expert Committee. World Health Organ Tech Rep Ser [Internet]. 1995;854:1–452. Disponível em: http://www.ncbi.nlm.nih.gov/pubmed/8594834
